# Higher Education Institutions as Strategic Centers for Promoting Social Innovation in Gerontology: Insights from the Senior Innovation Lab Training Initiative

**DOI:** 10.3390/geriatrics9030076

**Published:** 2024-06-08

**Authors:** Susana Feijóo-Quintas, Noelia Gerbaudo-González, Manuel Gandoy-Crego, Mª del Carmen Gutiérrez-Moar, Elísio Costa, David Facal

**Affiliations:** 1 Department of Developmental and Educational Psychology, University of Santiago de Compostela, 15782 Santiago de Compostela, Spain; susanafeijoo.quintas@usc.es; 2 Department of Psychiatry, Radiology, Public Health, Nursing and Medicine, University of Santiago de Compostela, 15782 Santiago de Compostela, Spain; noelia.gerbaudo@usc.es (N.G.-G.); manuel.gandoy@usc.es (M.G.-C.); 3 Department of Pedagogy and Learning, University of Santiago de Compostela, 15782 Santiago de Compopostela, Spain; mdelcarmen.gutierrez@usc.es; 4 Faculty of Pharmacy, CINTESIS@RISE, Competence Center on Active and Healthy Ageing (Porto4Ageing), University of Porto, 4050-313 Porto, Portugal; emcosta@ff.up.pt

**Keywords:** higher education institutions, social innovation, gerontology, entrepreneurship, competences

## Abstract

Background: Social innovation and gerontology develop a wide range of actions aimed at supporting and improving the needs of long-lived populations. Higher education institutions (HEIs) are drivers of change, and their potential to develop solutions through teaching students’ social innovation skills should be considered in the field of longevity. This article reports the results obtained by the Senior Innovation Lab (SIL) training initiative. Methods: Challenge-based learning, design thinking, and lean startup approaches were implemented in training 26 participants with the final aim of developing innovative solutions to previously identified long-lived population needs. Results: Final products were innovative ideas developed through collaboration between students, academic staff, and business employees, indicating the importance of adopting entrepreneurial approaches in academic teaching. The participants identified motivation and perseverance as the most relevant entrepreneurial skills and most of them also perceived that they possessed it. The participants also considered spotting opportunities (chance to add value) as the most desired skill throughout the whole experience. Conclusions: SIL’s assessment showed that social innovation methodologies contributed to the development of learning strategies, enabling potential solutions for the ageing challenges.

## 1. Introduction

Long-lived societies and their associated characteristics imply rethinking strategies for the challenges they pose. Social gerontology and psychogerontology must promote the search for alternative solutions, new methods, and cooperation between different sectors, such as the education, technology, and social sectors, to solve long-standing age-related problems of the population, with an enormous potential for innovation emerging [1,2].

Higher education institutions (HEIs) play two important roles in the field of gerontology. Training students in gerontology implies the transfer of knowledge about the field and its needs, guiding students to develop solutions to social problems through new learning methods [3,4,5,6,7]. In addition, through their mission to transform lives and inspire change, HEIs aspire to enable the development of stronger thinkers and stronger communities [3]. Evidence supports the importance of training in the use of entrepreneurial approaches [8] and the development of entrepreneurial and innovative skills [9,10] in higher education. Furthermore, it has been demonstrated that the student community perceives learning modules about entrepreneurship (understood as the development process of starting new initiatives or other types of proposals) and business development as basic requirements [11]. Considering that innovation results from a process characterized by two main features—a degree of newness and a degree of usefulness or success in application of something new [12]—the success of innovation in HEIs relies on their social networking capabilities, including how they collect resources, facilitate the knowledge dissemination process, and identify opportunities by forming social ties [13,14], enhancing collective actions and innovation processes.

Social innovation is defined as a group of new ideas with various forms such as products, services, processes, models, or regulations aimed at addressing social needs and fostering new relationships and collaborations [15,16,17]. In HEIs, socially innovation usually arises due to collective action and collaboration with institutional actors in the innovation ecosystems [18,19]. These innovation ecosystems are defined as collaborative arrangements through which different actors combine their individual offerings into a coherent solution for their customers [19]. HEIs can be drivers of change in innovation, and they should play a central role in promoting and creating social and economically sustainable benefits to the communities where they are located. Hence, the relationship between HEIs and innovation in the social field leads to social innovation.

Social innovation must consider changes in the profile of society and the adaptations that it demands, such as those that become specific characteristics of older adults. Likewise, innovative businesses, services, and activities aimed at the oldest members of the population should seek to satisfy the needs that emerge from increased longevity [20,21,22]. The fields of social innovation and gerontology are involved in developing a wide range of social and technological solutions that are implemented to support and improve home and organizational care services (nursing homes, hospitals) to address the needs of older adults. These initiatives are developed to improve daily living conditions in various areas (housing, communication, health, education) and in collaboration between organizations to meet their needs [23].

As Voorberg et al. [24] pointed out, older adults must be encouraged to become involved and actively participate in the process of social innovation. For this reason, the approach must consider that this segment of the population remains socially and economically relevant, and their involvement, as well as that of professionals in the gerontology sector, is fundamental in research and practice, adding value and enriching knowledge and research experience and skills. Thus, addressing the needs and preferences of older adults would be maximized [17,25,26,27].

The Senior Innovation Lab (SIL) training initiative emerged as a result of the above considerations, aiming to address the social need to highlight the social relevance of older adults in society, no longer as something pejorative but as an opportunity for social and economic growth. The SIL initiative seeks to favor the acquisition of innovation knowledge and tools to develop new solutions to different challenges and opportunities of older adults’ population, as well as to identify the importance and the need for social innovation for them. Its goals were as follows: (1) to promote knowledge and awareness in students and professors about the opportunities and challenges posed by the culture of innovation and social entrepreneurship, defined as a process that involves the innovative use and combination of resources to seek opportunities for stimulating social change and addressing social needs [28]; (2) to encourage the acquisition and development of entrepreneurial skills of the participants in the context of innovation; and (3) to facilitate an alliance between HEIs and participating organizations in the gerontology sector to promote knowledge transfer and enhance research and innovation capacities.

The current paper describes implementation of this pilot educational program in the field of older adults, implemented in the master’s degrees of the University of Santiago de Compostela (USC). The aim of the paper is to show how the SIL initiative applies innovation knowledge and tools to promote innovative solutions for the ageing challenges, promoting the understanding of how HEIs can enhance their training and networking capacities to facilitate the co-creation of social innovations in gerontology.

## 2. Materials and Methods

### 2.1. Participants

SIL is aimed at students, academic, and nonacademic staff of the master’s degrees offered by the USC. In this training initiative, a total of 26 people participated in the training (16 women, 62%; 12 men, 38%). Among them, 13 were master’s degree students (50%, age range from 20 to 30 years old), 9 were academic staff (35%, age range from 30 to 56), and 4 were nonacademic staff (15%, 30 to 45). The students who completed the training came from psychology, social work and sociology degrees. Three working groups were established in an attempt to balance the composition of the groups.

In addition, SIL was attended by 16 representatives of entities from the gerontology sector (12 women, 75%; 4 men, 25%). This group was formed by a panel of experts who provided feedback to the SIL participants on the final day of the course. The profile of the representatives varied according to the type of institution with which they were associated: care companies, nongovernmental organizations, public health system, and technology-based startups.

### 2.2. Activities

**Figure 1:**
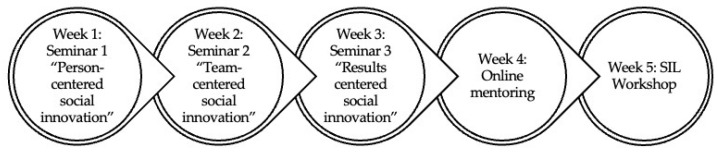
Schedule of the learning activities included in the Senior Innovation Lab.

**Figure 2:**
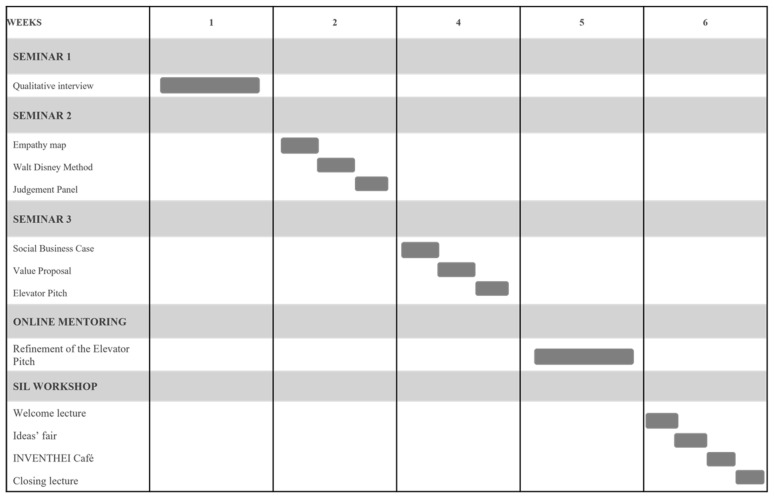
Learning milestones.

The SIL initiative is an educational experience consisting of a series of five weekly seminars. In this case, the series was formed by four on-site activities, each lasting three hours, and one online (support) activity, which lasted around two hours. The first three on-site sessions were seminars that were open to the university community (students, academic staff, and nonacademic staff). The fourth face-to-face session was a workshop, which was also attended by professionals and organizations from the field of gerontology (see Figure 1 and Figure 2).

To achieve the aims, agile innovation methodologies were applied, including challenge-based learning, design thinking, and lean startup. Challenge-based learning consists of responding to challenges of real life by creating solutions. This methodology works inversely to the traditional educational scheme, helping to ideate by generating multiple potential solutions [29]. There is evidence for the efficacy of this methodology in the training of innovative and entrepreneurial skills [8,30]. On the other hand, design thinking is a process used to find innovative solutions to a problem through understanding people’s needs. This methodology has five phases: empathize, define, ideate, prototype, and test [29,31]. Finally, lean startup involves validating the hypothesis during the process before arriving at the final solution. It consists of three phases: create/make, measure, and learn [32].

Seminar 1 “Person-centered social innovation” was the opening session of the SIL training initiative. The main aim of this activity was to highlight the importance of focusing social innovation on people’s needs, particularly the needs of a particular older adult. During this session, participants were introduced to the initiative and received training in social innovation, entrepreneurship, and the different methodologies and competences in this field. They learned about the societal challenges they were going to face, which had been selected by the coordinators of the master’s degrees in collaboration with external internship tutors and companies in the sector, in an unstructured way, through conversation. The data collected led to the identification of three challenges: (1) New technologies and ageing (How can technology be incorporated in nursing homes to improve the care processes that really matter? How can organizations in the sector be digitally transformed to offer quality care to people?); (2) Ageism and a change in the way we look at ageing (How can the population be made aware of the need to change the way older adults are viewed? How can age stereotypes be broken?) and (3) Models of care and social vision of older adults (How can truly personalized attention be provided? How can we move from effective care time to meaningful care time?). The participants were grouped into three teams, each tasked with addressing one of the challenges based on their preferences. The challenge-based learning methodology was implemented in this seminar. This involved analyzing different real-life obstacles to identify the challenges associated with older adults in the target population (in NW Spain). To achieve the principal objective of the seminar, the participants elaborated an older adult-specific profile that would be used to generate ideas for solutions to the proposed problems. Additionally, the design thinking approach was implemented, focusing on its first two phases: (a) “Empathize”, where the participants had to put themselves in the older adults’ place to understand their emotions, collect user’s requirements, and identify their needs by completing an empathy map; and (b) “Define”, where the participants refined the user’s requirements.

In the second seminar, “Team-centered social innovation” aimed to produce ideas or solutions to the proposed problems and the development of the chosen solution through teamwork. For this purpose, the third phase of the design thinking approach, “Ideate”, was implemented. Participants had to produce as many ideas as possible to meet the identified needs. Once they had conceived several potential solutions, the teams assessed each and chose the best one as the solution to be developed through the Social Business Case and the Value Proposal.

In the third seminar, “Results-centered social innovation” aimed to develop and disseminate the minimum viable product (MVP). To achieve this objective, the lean startup methodology was implemented. The participants were given the opportunity to learn theoretical knowledge about concepts such as MVP and elevator pitch. These concepts were put into practice through the first step of the lean startup cycle of phases: build–measure–learn. The participants developed the solutions (build) across the elaboration of the MVP, a product with the minimum features that will meet the identified needs, and the beginning of the development of the elevator pitch, a short, concise speech in which the product is presented attractively, and that would be presented at the final event (SIL Workshop).

Online mentoring was provided to support and guide participants in finishing and refining their elevator pitches. This type of communication aims to be clear, emotional, short, and impressive. Teams were guided to develop elevator pitches, including fundamental elements: (a) needs or problems to cover, (b) solutions provided, (c) main benefits, (d) nature as an ideal solution, and (e) call to action.

The SIL Workshop was the final, open session, designed to show the developed solutions during the seminars to the different organizations in the field and the other attendees. It took place two weeks after seminar three (Figure 2). During the activity, the two last phases of the lean startup approach, measure and learn, were implemented. The structure of this event included an Ideas Fair and an expert panel.

During the Ideas Fair, each team presented the elevator pitch to the audience, during five to ten minutes. This provided an opportunity to put the communication competences learned during the seminars into practice. At this stage, the participants completed the measurement of data and impact phases of the lean startup methodology. During the discussion with the expert panel, each team received feedback about the solution from representatives of the gerontology sector (i.e., “What recommendations would you give the team to move forward with the development of this solution?”). At this stage, the SIL participants completed the learning phase regarding the strengths and weaknesses of the solution, as well as its market possibilities. Further information about the learning activities developed can be found in Table S1 in Supplementary Materials.

### 2.3. Assessment

The SIL initiative was assessed through (A) trained entrepreneurial competences and (B) products/services proposed as solutions to the challenges addressed.

The SIL initiative adopted EntreComp, a comprehensive, flexible, multipurpose framework designed to support and inspire action to improve entrepreneurship [33]. This framework consists of three areas of competence that each include five competences: (1) Ideas and Opportunities: spotting opportunities, creativity, vision, valuing ideas, ethical and sustainable thinking; (2) Resources: self-awareness and self-efficacy, motivation and perseverance, mobilizing resources, financial and economic education, and mobilizing others; (3) Into Action: take the initiative; planning and management; coping with uncertainty, ambiguity and risk; working with others and learning through experience.

Based on previous evidence [8,9], the SIL initiative trained participants in seven key competences: (1) working with others, which is based on the ability to collaborate and teamwork to achieve the development of new solutions; (2) spotting opportunities, which refers to the identification of chances to create value through their abilities and skills by detecting the different older adults’ needs; (3) creativity, which is centered on the development of new or better solutions to the needs previously spotted, experiences with innovative approaches, and the combination of knowledge and resources to create value; (4) self-awareness and self-efficacy, which refer to the strengths and weaknesses, identification of the self and the group and at the same time the belief that they can achieve the aim; (5) motivation and perseverance, which are related to the need to remain concentrated and focused on what they want to achieve, as well as the need to keep trying and working on their ideas; (6) planning and management, which refer to the organization of the teamwork according to its priority, as well as defining goals and action plans; and (7) taking the initiative, which is related to assuming the lead and starting the creation value process to achieve its goals.

These entrepreneurial competences were assessed through a survey at the end of the SIL Workshop through online questionnaires designed to be completed quickly and concisely. Completion of the questionnaires was optional and anonymous. The three questions were aimed at determining how much the participants had learned about each entrepreneurial competence: (a) How relevant are the following competences for innovation?; (b) What innovation skills do you think you possess?; (c) Which of these seven innovation competences would you like to possess? The first question aimed to collect information on the competences that the attendees consider most relevant for entrepreneurship and innovation. The responses were scored on a five-point Likert scale: 1 “not relevant at all”; 2 “not very relevant”; 3 “indifferent”; 4 “relevant”; and 5 “very relevant”. The second question sought to collect data regarding the participants’ perception of their own competences in the field of innovation. The type of response requested was dichotomous (yes/no). The third question aimed to gather information on the desire to possess a series of competences in the field of innovation. The type of response requested was also dichotomous.

As already mentioned, the participants had to develop an innovative solution for the challenges addressed and present it at the final event through an elevator pitch. Each team received feedback on their proposed solutions from a specific panel formed according to the nature of the challenge presented.

The study was conducted following the current ethical standards. It was approved by the USC Bioethics Committee (code USC 61/2022).

## 3. Results

### 3.1. Entrepreneurial Competences

**Figure 3:**
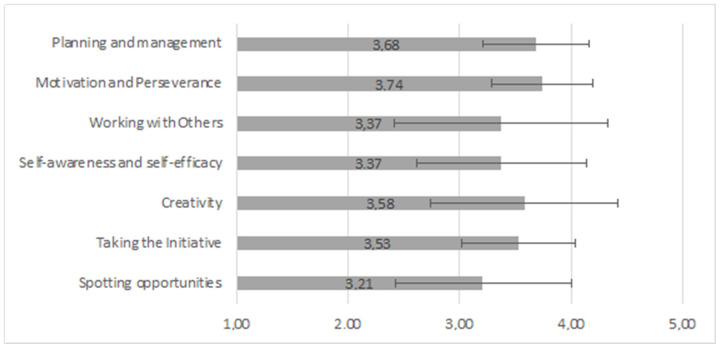
Responses to “How relevant are the following competences for innovation?”, with five-point Likert scale, with higher scores indicating higher perceived relevance and error bars showing standard deviations. *N* = 19 respondents.

An online survey was mailed to the 26 people who completed the training. The total number of responses received was 19 (i.e., 73% of the participants). The responses to the first question, about the relevance of entrepreneurial competences for innovation, are shown in Figure 3.

The participants considered the competences important/very important for innovation. The most outstanding was “Motivation and Perseverance” and the least outstanding was “Spotting opportunities”. There was only a slight variability (SD 0.45) in responses, indicating that “Motivation and Perseverance” was the most important competence for the participants. However, in the case of the least important (“Spotting opportunities”), there was greater variability (SD 0.79), indicating differences in its perceived importance in the social innovation field.

**Figure 4:**
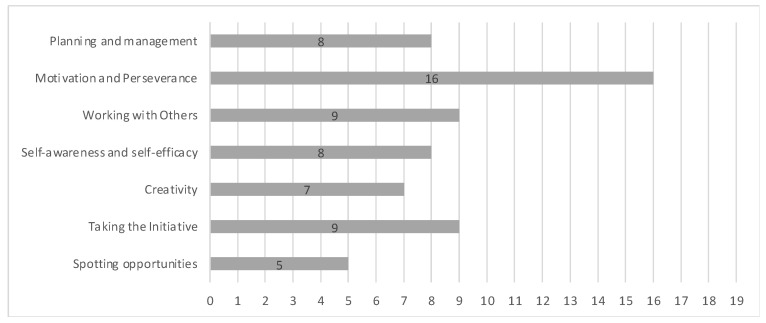
Responses to “What innovation skills do you think you have?“, with numbers corresponding to Yes responses. *N* = 19 respondents.

The proportions of responses to the second question are shown in Figure 4. Most of the participants who responded to the survey perceived themselves as having the competence of “Motivation and perseverance”. In addition, about 50% of the participants perceived themselves as having the ability to collaborate and work with others as a group (“Working with others”), as well as to have the courage to take the lead (“Taking the Initiative”). Moreover, 42% of the participants perceived themselves to have skills in defining goals and action plans (“Planning and management”), and identifying their strengths and weaknesses (“Self-awareness and self-efficacy”). On the other hand, only 26% of the participants perceived that they had the ability to detect opportunities for innovation (“Spotting opportunities”).

**Figure 5:**
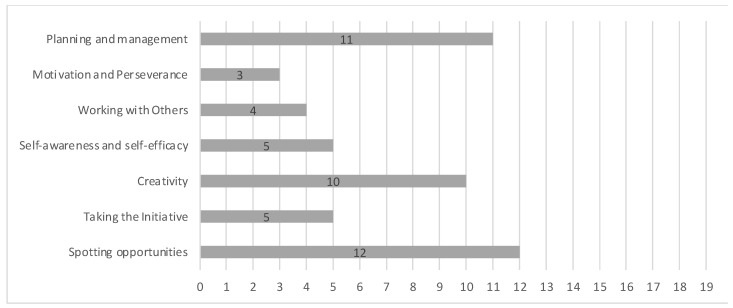
Responses to “Which of these seven innovation competences would you like to have?“, with numbers corresponding to Yes responses. *N* = 19 respondents.

The proportions of responses to the third question are shown in Figure 5. It should be noted that despite having been identified as the least acquired competence, 63% of the participants considered that they would like to be able to spot opportunities for innovation.

### 3.2. Innovation Solutions

**Figure 6:**
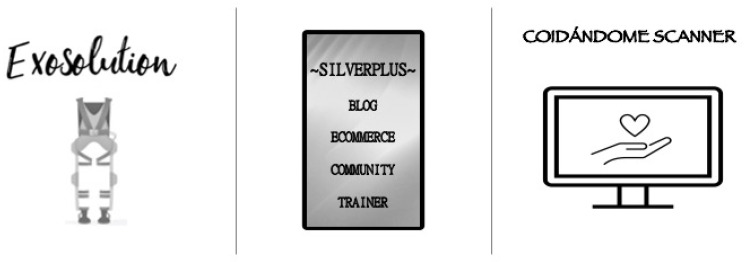
Logos of the three solutions developed through the Senior Innovation Lab training initiative: *Exosolution*—use of an ergonomic exoskeleton structure to maintain social life by enhancing strength and mobility; *Silver Plus*—digital platform designed for recently retired older adults who seek to socialize, in order to prevent situations of unwanted loneliness; *Coidandome Scanner*—digital platform designed to facilitate searches for services that offer person-centered care.

At the end of the training activities, each group presented an innovative solution aimed at satisfying a specific need related to the challenge in question (Figure 6), the initial formulation of the minimum viable product, and an elevator pitch. The expert panel, made up of members from companies, associations, and foundations, provided feedback after each short presentation. In this way, the experts’ feedback did not condition the development of the innovative ideas but promoted improvements considering the actual social needs and previous experiences of the entrepreneurs. The comments received and the main characteristics of the solutions presented are summarized below.

Group one worked on a challenge involving new technologies and ageing. After profiling the user and detecting their needs, they came up with a solution: an ergonomic and adaptable structure named “Exosolution”. The elevator pitch revealed that the product was designed for people with mobility problems who want to recover their mobility to maintain their social life. The solution was an ergonomic exoskeleton structure that allows the recipient to move autonomously. The structure was accompanied by a wrist device that guides its operation in a simple and intuitive way, thanks to an integrated voice control that maintains the person connected with chosen contacts via calls and video. The feedback from experts yielded the following recommendations: (a) to develop a visual example of the product; (b) to further investigate the objectives proposed by the solution; (c) to strengthen the communication strategy; (d) to involve stakeholders from the beginning in the development of the solution; (e) to carry out a benchmarking activity to learn about other similar products/projects.

Group two addressed a challenge related to ageism and considered how the general population can be made aware of the need to change the way older adults are viewed. The group proposed a digital platform as a solution and named it “Silver Plus”. The elevator pitch revealed that the solution was designed for recently retired older adults who seek to socialize more and find spaces and people with shared interests. It included a portfolio of services and personalized advice: a blog that addresses issues of interest to the target group; a virtual store that includes different products and services specially designed for them, and access to a community network for invitations to participate in events, activities, and trips; personalized advisory service (“silver trainer”) where each case is studied and a personalized plan is developed; discounts for activities, events and trips, local shops, and more, would also be offered. The experts considered the proposed platform an adequate solution to prevent situations of unwanted loneliness. The experts’ recommendations can be summarized as follows: (a) to reassess the age segment at which the product is targeted; (b) to evaluate the applicability of the platform in rural areas; (c) to study how digital skills affect product demand; and (d) to establish alliances with strategic partners.

Group three dealt with the challenge of new models of care and they considered how to provide truly personalized attention, and how to transition from effective care time to meaningful care time. The group proposed a digital platform as a solution, which they named “Coidándome” (Taking Care Of Me) Scanner. The elevator pitch showed that the platform was designed to facilitate searches for services that offer personalized attention to older adults. The panel of experts highlighted that the proposed solution allows creation of a network, which is key to addressing the challenge. One of the strengths highlighted was that the proposed solution aims to move forward in line with person-centered care. The recommendations received can be summarized as follows: (a) to extend the services included in the application to more users; (b) to incorporate the opinions and experiences of professionals, current users, and family members; (c) to propose a business model that considers the perspective of professionals and companies in the sector; and (d) to request calls for grants and aid to carry out the project.

## 4. Discussion

The aim of this training was to transmit knowledge and tools for social innovation and favor collaboration between the participants in formulating solutions to the proposed challenges, reinforcing the role of HEIs in the generation of applied knowledge in the gerontology sector. It is worth highlighting the role that companies played, ranging from formulation of the challenges to the feedback regarding the solutions designed by the participants and what the participants learned. It allowed linking the participants’ proposals with the social and local reality.

In this sense, it is worth noting how HEI initiatives (such as SIL) can facilitate the transfer of knowledge from the business community to the student body, paving the way for innovation and the retransfer of that knowledge to society. These findings further support the idea that to create successful innovation, HEIs depend on their social networking capabilities such as identifying opportunities by forming social bonds, thus increasing legitimacy for collective action and social innovation process ties [13,14].

The experience during the SIL project shows how HEIs can play a pivotal role as network facilitators and mediators that improve the connectivity between the actors to stimulate innovation [7]. Previous studies have also demonstrated that social innovation in HEIs usually arises because of collective action and collaboration with institutional actors of the innovation ecosystem [7,18,19].

The experience during the SIL initiative shows the benefits of including social innovation methods in HEI learning strategies. The participants produced innovative solution proposals to specific social challenges after only four training sessions. It thus appears that the participants managed to appropriate the tools and strategies taught. Using the challenge-based learning, design thinking, and lean startup approaches enabled the participants to respond to the challenges of the gerontology sector by creating innovative solutions and validating their hypotheses before reaching the final proposal. The results obtained provide support for the benefits of embodying principles of social innovation across different curricula [2,3].

Only seven of the fifteen EntreComp competences were selected for inclusion in the SIL training initiative with the challenge-based approach. This was also performed by Colombelli et al. [8] and Ferreras-García et al. [9] due to the type of training or study, although Garbut et al. [10] and Ņikitina et al. [34] used the entire set of competences. In the present case, the selection was justified by the short length of the trial, only three training sessions, and the pilot nature of the training. This feature would be considered in the development of the future editions of this initiative, which are already underway.

The “Spotting opportunities” competence was the most desired, although it was initially identified as the least important, contrary to the findings of Ferreras-García et al. [9] and Ņikitina et al. [34], who found that this was one of the most relevant competences but perceived as the least commonly possessed by the participants. The differences in results may be due to the dynamics of the training, as the opportunities that could be identified, i.e., the challenges, were already presented to the participants. According to the Entrecomp model [33], “spotting opportunities” refers to the competence to identify and seize opportunities to add social, cultural, and economic value; to assess whether these opportunities can be attractive, feasible, and successful; and to establish new connections and bring together elements of the ecosystem to create opportunities to create value. In this respect, the competence to “identify opportunities” is central to enable innovative solutions to societal challenges in a changing context, not only in terms of economic and technological development, but also in terms of the demographic characteristics and age of its participants.

Our educational process has several limitations that should be taken into consideration. Firstly, the SIL initiative was aimed at master’s degrees students at the university. This implied that the profile of the participants was relatively homogeneous and restricted, made up mostly of graduates in social and behavioral sciences. In addition, the initiative was not open to undergraduate or doctoral students. Both factors can be considered limitations as it was not possible to evaluate the potential of interdisciplinary work among students during training. Based on the proposed solutions, collaboration between fields of knowledge more closely associated with economics and engineering, for example, could nurture ideas.

On the other hand, the SIL initiative could have benefited from an intergenerational co-design approach. This perspective is more appropriate when innovating in the gerontology sector, as it involves continuous and collaborative work with the target population of the solutions.

Finally, limitations in the study’s design include the lack of a control group to assess their perception of innovative competences in absence of specific training. Additionally, there was a lack of continuous assessment of the training process and its intermediate products. Complementary approaches are considered, such as using “tickets out of class” responding to questions about learnings and questions that participants take away from each session.

## 5. Conclusions

This article reports the results obtained after the first SIL initiative. The main goal of this study was to report the contributions of the initiative to the field of social innovation in gerontology. Senior innovative lab training may engender creative learning strategies and enable potential solutions for the ageing challenges, providing tools and knowledge to improve an ageing society.

Complementarily, this study explores the role of HEIs in promoting the understanding of how HEIs can facilitate transference of knowledge in the field of gerontology. As stakeholders in society, HEIs can use their networking capabilities to facilitate and promote successful innovations. Thus, companies were invited to participate from the beginning, formulating the challenges, and finally providing feedback on the solutions designed by the participants.

Notwithstanding some limitations, the study findings suggests that social innovation skills should be taught in the training curricula of the new generations of professionals in the sector of gerontology.

## Data Availability

The original contributions presented in the study are included in the article; further inquiries can be directed to the corresponding authors.

## Supplementary Materials

The following supporting information can be downloaded at: https://www.mdpi.com/article/10.3390/geriatrics9030076/s1, Table S1: Learning activities developed.
