# Supplementary material for: Higher Education Institutions as Strategic Centers for Promoting Social Innovation in Gerontology: Insights from the Senior Innovation Lab Training Initiative
Source: Geriatrics (Basel). 2024 Jun 8;9(3):76. doi: 10.3390/geriatrics9030076 (PMC11202931; doi:10.3390/geriatrics9030076)
Supplement: Supplementary file 1 [file geriatrics-09-00076-s001.zip › geriatrics-2998603-supplementary.pdf]

**Table S1.** Learning activities developed.

| Session | Activity | Aim | Competences trained |
|---|---|---|---|
| Seminar 1 | Interview: participants were divided in three groups according to the challenge of their choice and trained to implement the interview. Steps:<br>Interview preparation<br>Interview itself to empathize<br>Share and extract learnings | Motivating cooperation and collaboration among participants to seek solutions to specific challenges. | - Spotting opportunities<br>- Working with others |
| Seminar 2 | Empathy map: the participants had to answered questions related with the older adult for whom they were designing the solution that appear in this tool.<br>Steps:<br>- Definition of the ideal user person.<br>- Completion of the empathy map.<br>- Reformulation of the challenge based on learning. | Understand the people for whom we want do design the solution. | - Creativity<br>- Self-awareness and self-efficacy<br>- Taking the initiative |
| | Walt Disney Method: the participants used this technique to create and generate new ideas and solutions.<br>Steps:<br>- Propose dreamlike or impossible ideas.<br>- How the proposed ideas can be carried out, adopting a more realistic view.<br>- Identify which of the ideas suits best the identified needs. | Development of creative ideas. | - Creativity<br>- Self-awareness and self-efficacy<br>- Taking the initiative |
| | Judgement panel: it consisted in the identification of ideas' pros, cons, deficiencies and obstacles. | The participants show their ability to see both the positive and negative qualities of the ideas and apply them or discard them in favor of another that may have better development possibilities | - Self-awareness and self-efficacy<br>- Taking the initiative |
| Seminar 3 | Social Business Case: the participants had to answer a series of questions related with the idea:<br>- Business model<br>- Pilot budget<br>- Economical impact<br>- Personal impact<br>- Environmental impact | Development and validation of the selected ideas. | - Planning and management |
| | Value Proposal: it consisted in answering the following questions about the idea: | Development and validation of the selected ideas | - Motivation and perseverance |

| | | | |
|---|---|---|---|
| | - What do you offer? (Functions and components)<br>- Benefits of the idea/ What for?<br>- Costs and effort of the idea | | - Planning and management |
| | Elevator Pitch: consisted in the creation and development of a speech where the participants could sell their idea to the market. | Communicate effectively the proposal | - Motivation and perseverance<br>- Planning and management |
| Online mentoring | Refinement of the Elevator Pitch: consisted in the improvement of each teams' speech by presenting them to the other participants and taking into account their suggestions and the feedback from the mentor. | Communicate effectively the proposal | - Self-awareness and self-efficacy<br>- Working with others |
| Workshop | Welcome lecture: "Improving innovation through collaborative work". It was focused on how the phenomenon of longevity challenges innovation considering the ecosystem and stakeholders involved. | Encourage students to innovate in gerontological field. | - Self-awareness and self-efficacy<br>- Motivation and perseverance |
| | Ideas' fair: presentation of the Elevator Pitches in front of the audience. | Presentation of the innovative ideas developed.<br>Put into practice the skills trained during the initiative. | - Self-awareness and self-efficacy |
| | INVENTHEI Café: it consisted in a participatory debate organized in worktables. | Receive feedback from a panel of experts. | - Self-awareness and self-efficacy |
| | Closing lecture: "Innovation in the socio-health field. Learned lessons". It was focused in show example of a local social innovation company. | To inspire the attendees to follow the path of entrepreneurship and social innovation. | - Motivation and perseverance |
